# For Good Roads

**Published:** 1897-03

**Authors:** 


### FOR GOOD ROADS.

Senator Brown's proposed legislation for good roads in Pennsylvania means much to the veterinary profession. It will encourage more driving; it will enhance the value of horses; it will stimulate the breeding of a high class of roadsters; it will create a larger market for saddle horses. In fact, it has everything to commend it, and is the evidence of the highest type of advanced civilization. It therefore places a duty before every member of the profession to aid this movement through our Legislature, and our approval is only half performed until we have written our district representatives at Harrisburg in favor of this measure. These are times of labor, and you must be up and doing all the time; for every good and wise law that will add a measure of prosperity, of pleasure, of profit, and contribute to the well-being of the general public is ours to aid and support.

